# Supplementary material for: Genome-wide association study in quinoa reveals selection pattern typical for crops with a short breeding history
Source: eLife. 2022 Jul 8;11:e66873. doi: 10.7554/eLife.66873 (PMC9388097; doi:10.7554/eLife.66873)
Supplement: Figure 3—source data 4. — The red horizontal line indicates the significant threshold (Bonferroni correction) -log10(1.67e-8). [file elife-66873-fig3-data4.pdf]

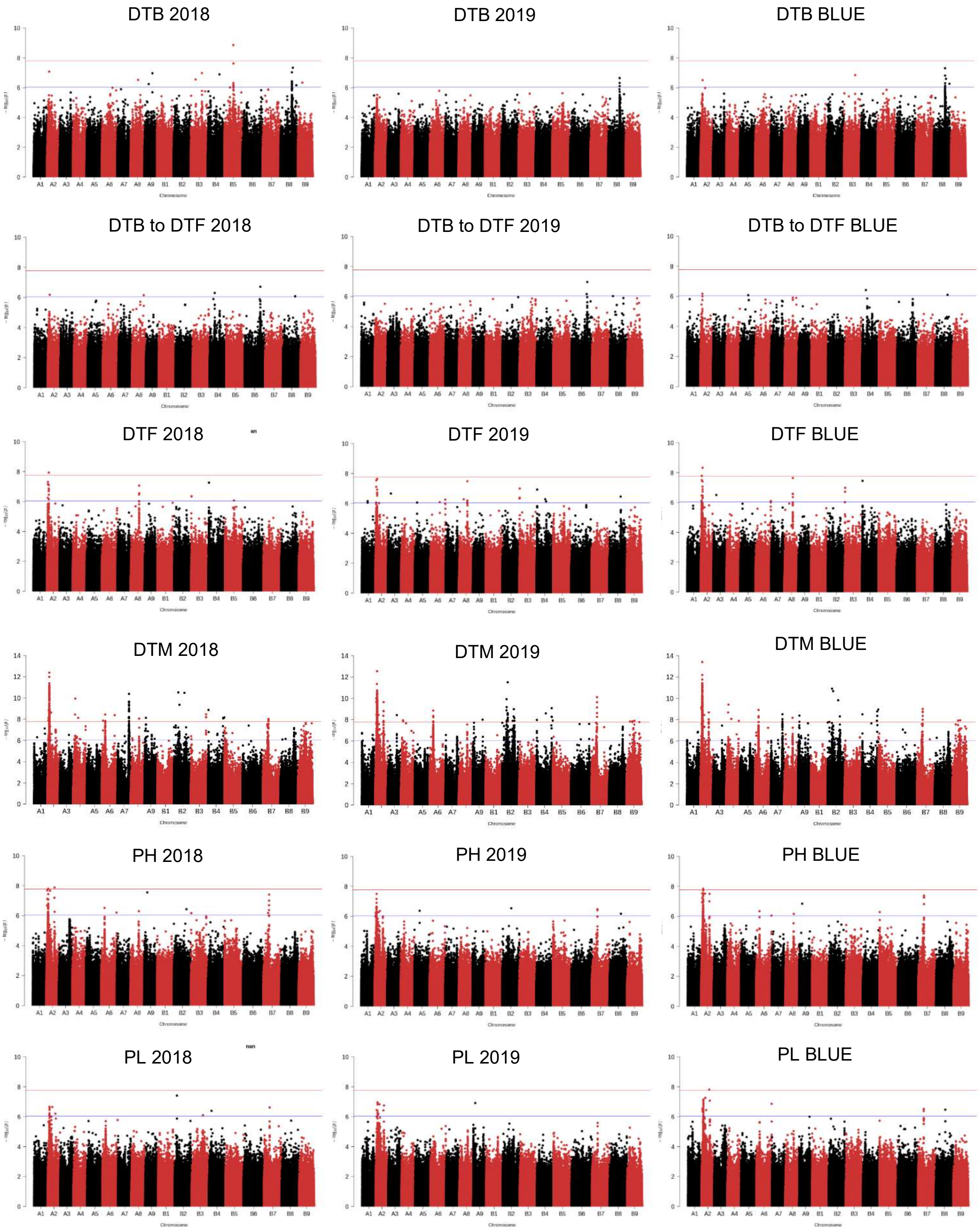

Figure 3-source data 4: *cont*.

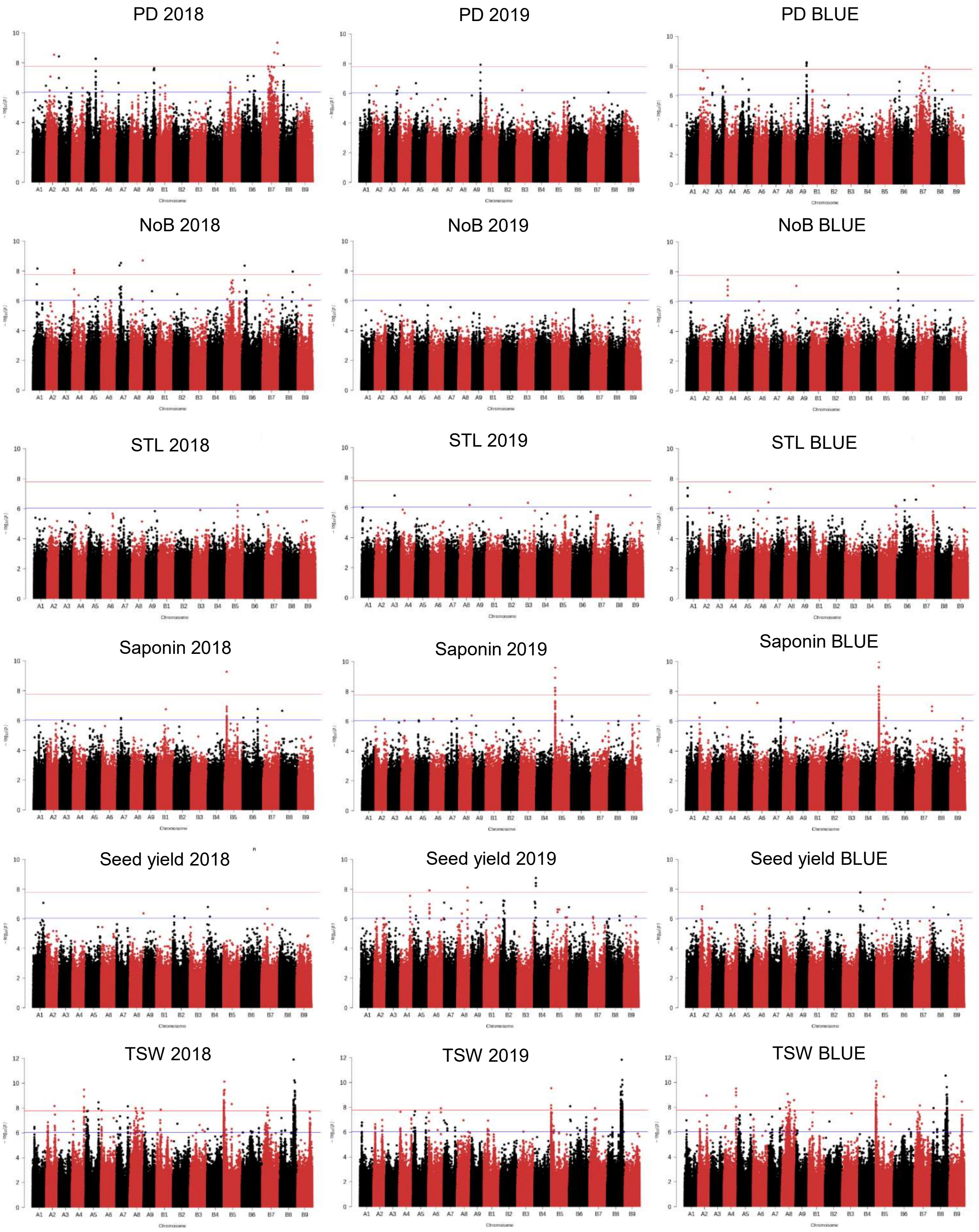

Figure 3-source data 4: *cont.*

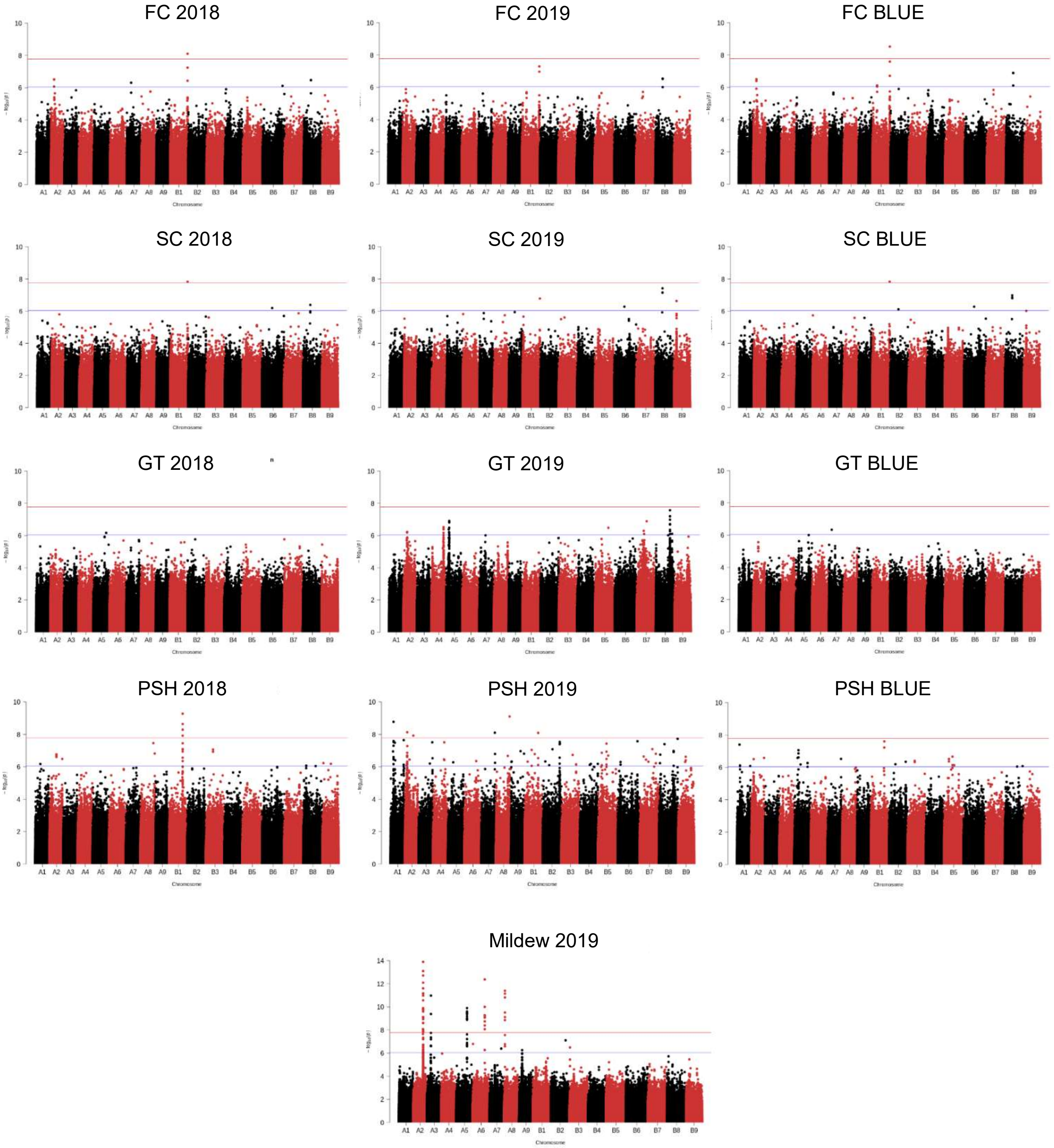

Figure 3-source data 4: Manhattan plots from GWAS with data from 2018 (left), 2019 (center), and the mean of both years (right): The blue horizontal line indicates the suggestive threshold -log10 (8.98E-7). The red horizontal line indicates the significant threshold (Bonferroni correction) -log10(1.67e-8).
