# Supplementary material for: Genome-wide association study in quinoa reveals selection pattern typical for crops with a short breeding history
Source: eLife. 2022 Jul 8;11:e66873. doi: 10.7554/eLife.66873 (PMC9388097; doi:10.7554/eLife.66873)
Supplement: Figure 3—source data 5. [file elife-66873-fig3-data5.pdf]

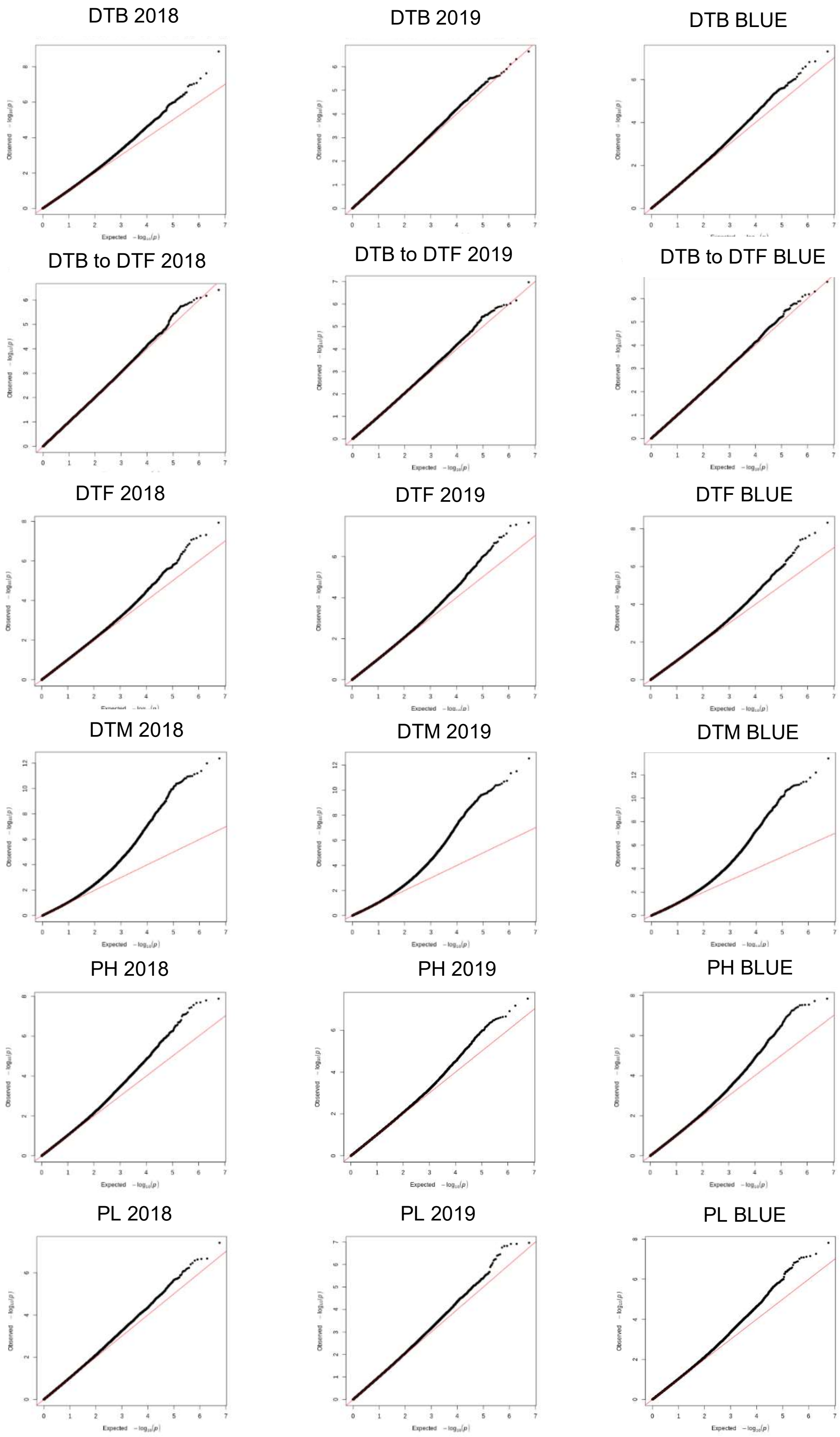

Figure 3-source data 5: *cont.*

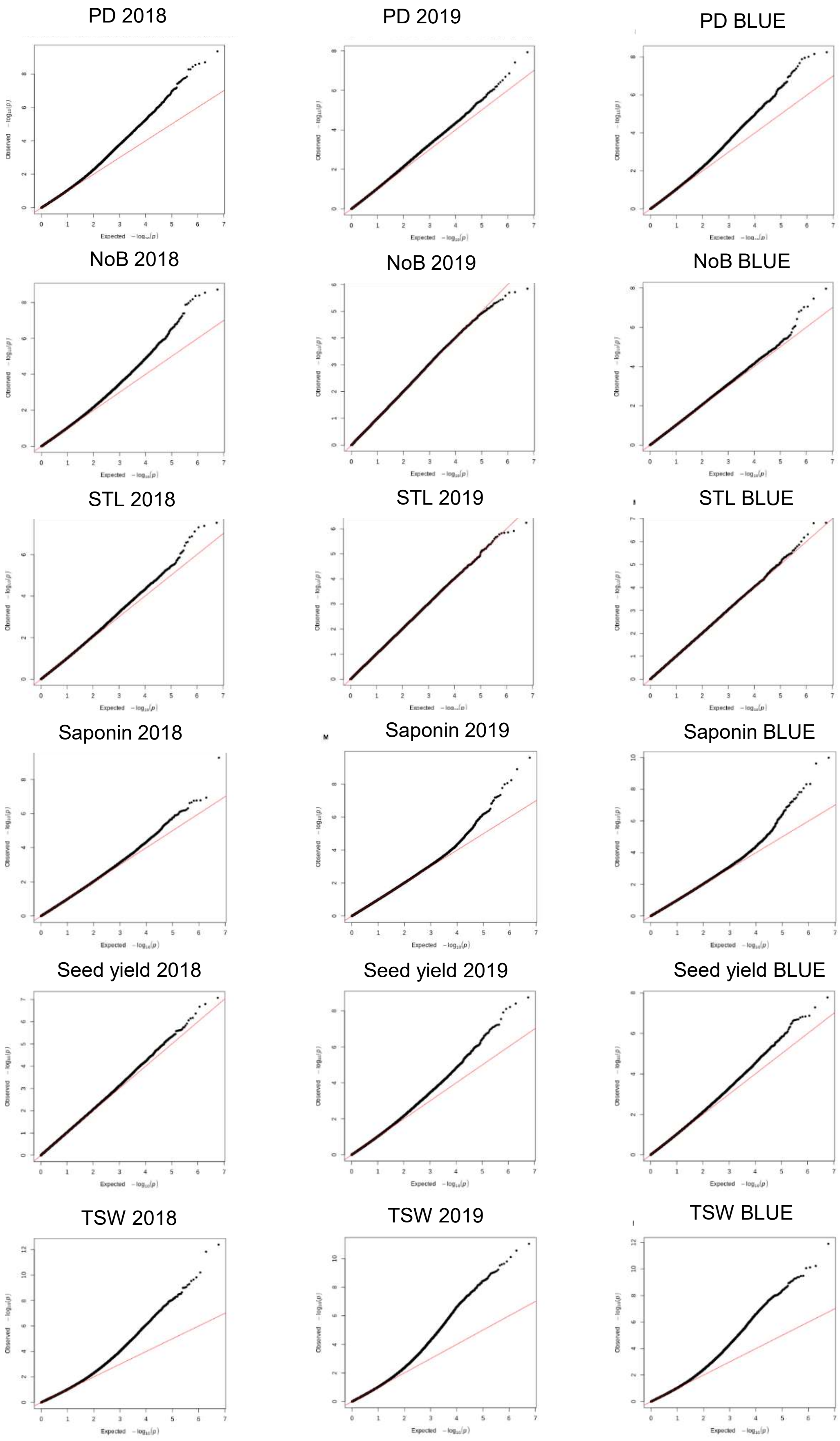

Figure 3-source data 5: *cont.*

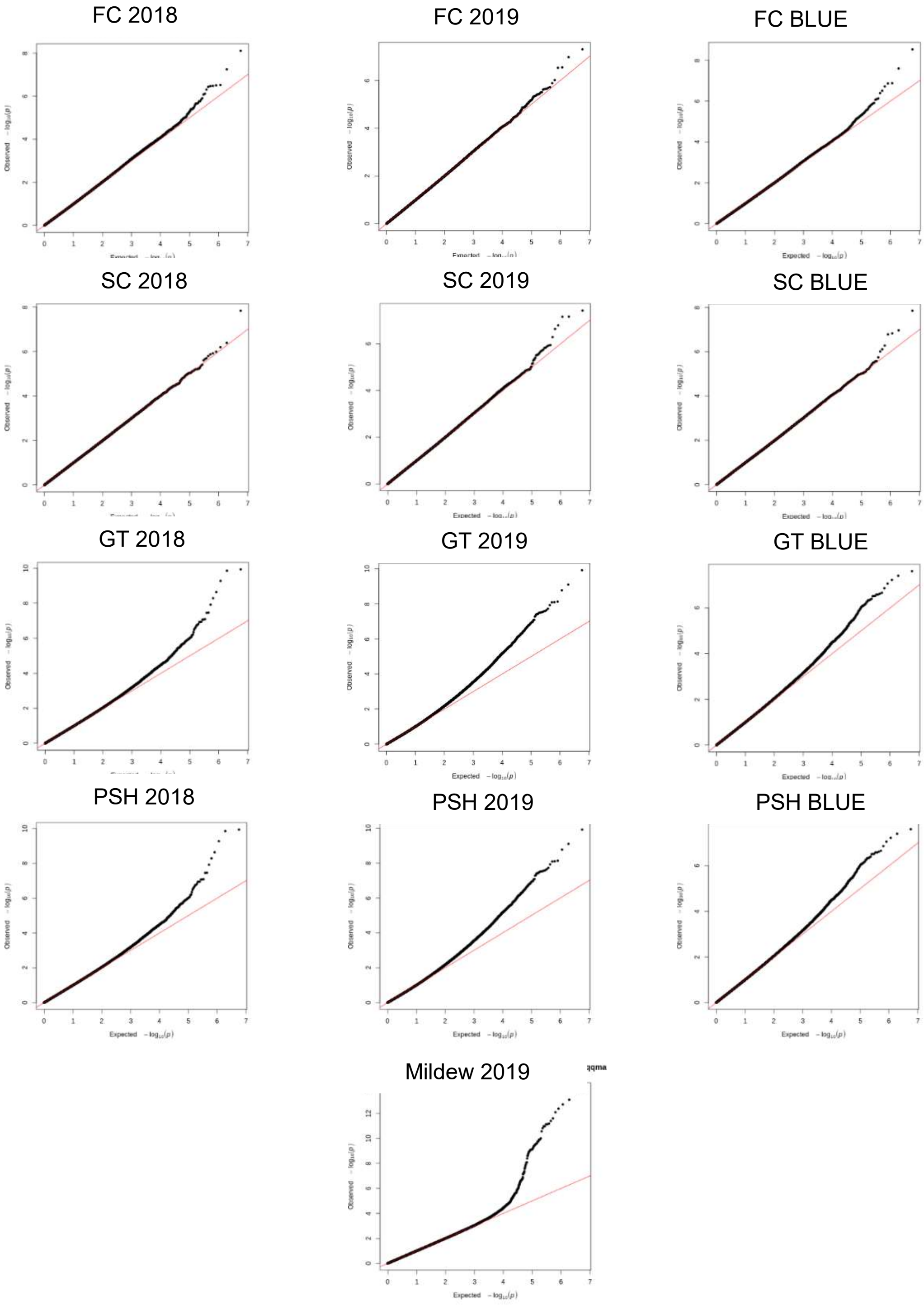

Figure 3-source data 5: Quantile-quantile plots of GWAS in two years, 2018 (left) and 2019 (center), and best linear unbiased estimates (right).
